# Diagnostic challenge of pulmonary embolism during testosterone replacement therapy for primary hypogonadism

**DOI:** 10.1210/jcemcr/luag312

**Published:** 2026-10-13

**Authors:** Hamza El Hadi, Silke Dorothea Hölscher, Jonel Trebicka, Elena Vorona

**Affiliations:** Department of Medicine B for Gastroenterology, Hepatology, Endocrinology and Clinical Infectiology, University Hospital Muenster, 48149 Muenster, Germany; Department of Medicine B for Gastroenterology, Hepatology, Endocrinology and Clinical Infectiology, University Hospital Muenster, 48149 Muenster, Germany; Department of Medicine B for Gastroenterology, Hepatology, Endocrinology and Clinical Infectiology, University Hospital Muenster, 48149 Muenster, Germany; Department of Medicine B for Gastroenterology, Hepatology, Endocrinology and Clinical Infectiology, University Hospital Muenster, 48149 Muenster, Germany

**Keywords:** testosterone replacement therapy, pulmonary embolism, male hypogonadism

## Abstract

Testosterone replacement therapy (TRT) is widely used in the management of male hypogonadism to restore physiological androgen levels and improve related symptoms. Although generally considered safe under appropriate monitoring, its association with pulmonary embolism (PE) remains controversial. We report a case of a 50-year-old man who independently and progressively shortened testosterone injection intervals due to recurrent hypogonadal symptoms. Despite the absence of overt cardiopulmonary symptoms, diagnostic workup revealed bilateral PE. This case highlights an atypical presentation of PE in a male patient receiving TRT, in whom nonspecific symptoms such as fatigue and reduced exercise tolerance were initially attributed to hypogonadism, potentially masking an underlying life-threatening thromboembolic event. It underscores the importance of periodic reassessment of TRT, clinical vigilance for thrombotic complications, and improved risk-stratification tools to guide monitoring and management.

## Introduction

Testosterone replacement therapy (TRT) is a well-recognized approach in men with primary hypogonadism to maintain secondary sex characteristics and improve clinical features consistent with androgen deficiency such as reduced libido, erectile dysfunction, fatigue, and physical strength [1]. The beneficial effects of treatment may take up to 12 months to become apparent [1]. A failure to achieve sufficient improvement of clinical hypogonadal symptoms requires a prompt reevaluation of administration route, dosage, adherence, and serum testosterone levels [2].

In addition to its beneficial effects, patients under TRT should be regularly monitored for adverse effects by assessing testosterone concentration, hematocrit, cardiovascular, and urological profile in order to ensure safety and efficacy [3]. In contrast to its wide range of benefits in hypogonadal men, conflicting data exist regarding the incidence and early detection of venous thromboembolism (VTE) associated with TRT [4].

Venous thromboembolism, mainly acute pulmonary embolism (PE), is considered a major contributor to the global disease burden due to its association with high morbidity and mortality. Major known conventional risk factors in general population contributing to PE include advanced age, active malignancy, immobility, and hypercoagulation disorders. To date, there is no validated risk-stratification system to predict PE in hypogonadal men receiving TRT.

Case reports have documented the occurrence of PE during TRT, with marked heterogeneity in clinical presentation, individual risk factors, and diagnostic approaches [5-7]. Herein, we present a case of a 50-year-old male on TRT who was incidentally diagnosed with bilateral segmental and subsegmental PE. This case highlights the need to identify high-risk individuals receiving TRT and to promptly differentiate hypogonadal symptoms from other conditions with overlapping clinical features.

## Case presentation

A 50-year-old male patient presented for the first time at our endocrinology outpatient clinic for clinical follow-up of previously diagnosed primary hypogonadism. His medical history was significant for unilateral orchiectomy performed 22 years earlier for a nonseminomatous testicular tumor. A detailed retrospective review of the available medical records additionally revealed that the patient had subsequently received 4 cycles of bleomycin, etoposide, and cisplatin (BEP) chemotherapy as adjuvant treatment. The patient denied nicotine, alcohol, or illicit drug use and reported no regular medications other than TRT.

The patient has been receiving TRT in form of testosterone enanthate (250 mg) administered every 4 weeks. During the clinical interview, he reported that, over the preceding 8 weeks, he had independently and progressively shortened the injection intervals because of recurrent symptoms attributed to hypogonadism, including fatigue, reduced libido, and reduced exercise tolerance. At the time of presentation, he was self-administering one-quarter of a 250 mg ampoule (62.5 mg testosterone enanthate) every 2 days. Despite this substantial modification of his testosterone regimen, his symptoms, particularly fatigue, had not improved.

## Diagnostic assessment

On examination, he was afebrile and tachycardic (100 beats/minute), with normal respiratory rate. Lung auscultation was clear bilaterally, and cardiac examination demonstrated regular rhythm. There was no evidence of jugular venous distention or peripheral edema. The physical examination was otherwise unremarkable. His body mass index was 25 kg/m^2^.

Initial laboratory testing revealed a marked elevation of hemoglobin (Hb) (18.2 g/dL [182 g/L]; reference range: 13.5-16.9 g/dL [International System of Units (SI): 135-169 g/L]) and an increased hematocrit (53.4% [0.534 L/L]; reference range: 40-49% [SI: 0.40-0.49 L/L]). C-reactive protein (CRP) was mildly elevated at 4 mg/dL (40 mg/L; reference range: <0.5 mg/dL [SI: <5 mg/L]).

Furthermore, a markedly elevated serum testosterone level was observed (15 ng/mL [52.0 nmol/L]; reference range: 1.9-7.4 ng/mL [6.6-25.7 nmol/L]). The last testosterone injection (testosterone enanthate 250 mg) had been administered 2 days prior to blood sampling. Laboratory findings were summarized in Table 1.

**Table 1:** Baseline and follow-up laboratory findings

Variable	Reference range	At admission	4 months after cessation of TRT
Hb	13.5 to 16.9 g/dL (135-169 g/L)	18.2 g/dL (182 g/L)	16 g/dL (160 g/L)
Hematocrit	40% to 49% (0.40-0.49 L/L)	53.4% (0.534 L/L)	49% (0.49 L/L)
White-cell count	3.9 to 10.9 × 10^3^/μL (3.9-10.9 × 10^9^/L)	11.9 × 10^3^/μL 11.9 × 10^9^/L	5.6 × 10^3^/μL 5.6 × 10^9^/L
Sodium	132 to 145 mEq/L (132-145 mmol/L)	140 mEq/L (140 mmol/L)	142 mEq/L (142 mmol/L)
Potassium	3.5 to 4.8 mEq/L (3.5-4.8 mmol/L)	4.1 mEq/L (4.1 mmol/L)	4.4 mEq/L (4.4 mmol/L)
Creatinine	<1.4 mg/dL (<123.8 μmol/L)	1.2 mg/dL (106.1 μmol/L)	1.2 mg/dL (106.1 μmol/L)
ALT	10 to 50 U/L (0.17-0.83 µkat/L)	32 U/L (0.53 µkat/L)	29 U/L (0.48 µkat/L)
AST	10 to 50 U/L (0.17-0.83 µkat/L)	29 U/L (0.484 µkat/L)	29 U/L (0.484 µkat/L)
HbA1c	4.3% to 6.1% (23-43 mmol/mol)	5.2% (33 mmol/mol)	—
Total cholesterol	<200 mg/dL (<5.18 mmol/L)	222 mg/dL (5.75 mmol/L)	—
LDL cholesterol	<130 mg/dL (<3.37 mmol/L)	157 mg/dL (4.07 mmol/L)	—
HDL cholesterol	>40 mg/dL (>1.04 mmol/L)	55 mg/dL (1.42 mmol/L)	—
Triglycerides	<150 mg/dL (<1.70 mmol/L)	120 mg/dL (1.36 mmol/L)	—
TSH	0.2 to 4.2 μIU/mL (0.2-4.2 mIU/L)	2.3 μIU/mL (2.3 mIU/L)	—
Testosterone	1.9 to 7.4 ng/mL (6.6-25.7 nmol/L)	15 ng/mL (52.0 nmol/L)	3.9 ng/mL (13.5 nmol/L)
D-dimer	<0.4 μg/mL (<0.4 mg/L)	16.9 μg/mL (16.9 mg/L)	
CRP	<0.5 mg/dL (<5 mg/L)	4 mg/dL (40 mg/L)	—
Factor II activity	>70%	128%	—
Factor V activity	>70%	108%	—
Factor X activity	>70%	104%	—
Factor VIII activity	>70%	196%	—
Factor IX activity	>70%	103%	—
Factor XI activity	>70%	98%	—
Factor XII activity	>70%	109%	—
Factor XIII activity	>70%	148%	—
Protein C activity	70% to 140%	108%	—
Protein S	60% to 140%	81%	—
Anticardiolipin IgG	<10 U/mL	0.8 U/mL	—
Anticardiolipin IgM	<7 U/mL	0.1 U/mL	—
β2-glycoprotein IgG	<8 U/mL	0.4 U/mL	—
β2-glycoprotein IgM	<8 U/mL	0.1 U/mL	—
Factor V mutation		Negative	—
Prothrombin mutation		Negative	—

Values in parentheses are International System of Units (SI). For analytes for which the conventional and SI units are identical or no distinct SI conversion is routinely used, values are reported once.

Abbreviations: ALT, alanine aminotransferase; AST, aspartate aminotransferase; CRP, C-reactive protein; Hb, hemoglobin; HbA1c, glycated hemoglobin; HDL, high-density lipoprotein; Ig, immunoglobulin; LDL, low-density lipoprotein; TRT, testosterone replacement therapy; TSH, thyroid-stimulating hormone.

Given this laboratory constellation and an intermediate clinical suspicion of a possible thromboembolic event, particularly in the context of remarked elevated hematocrit, tachycardia, and reported fatigue, D-dimer testing was performed to further evaluate and exclude this diagnosis.

The D-dimer level was markedly elevated at 16.9 mg/L (16.9 µg/mL) (reference range: <0.4 mg/L [SI: <0.4 µg/mL]).

In light of these clinical and laboratory findings, an urgent contrast-enhanced computed tomography (CT) scan of the thorax was performed, revealing bilateral segmental and subsegmental PE (Fig. 1). In addition, imaging findings suggested the presence of a left-sided pulmonary infarction.

**Figure 1:**
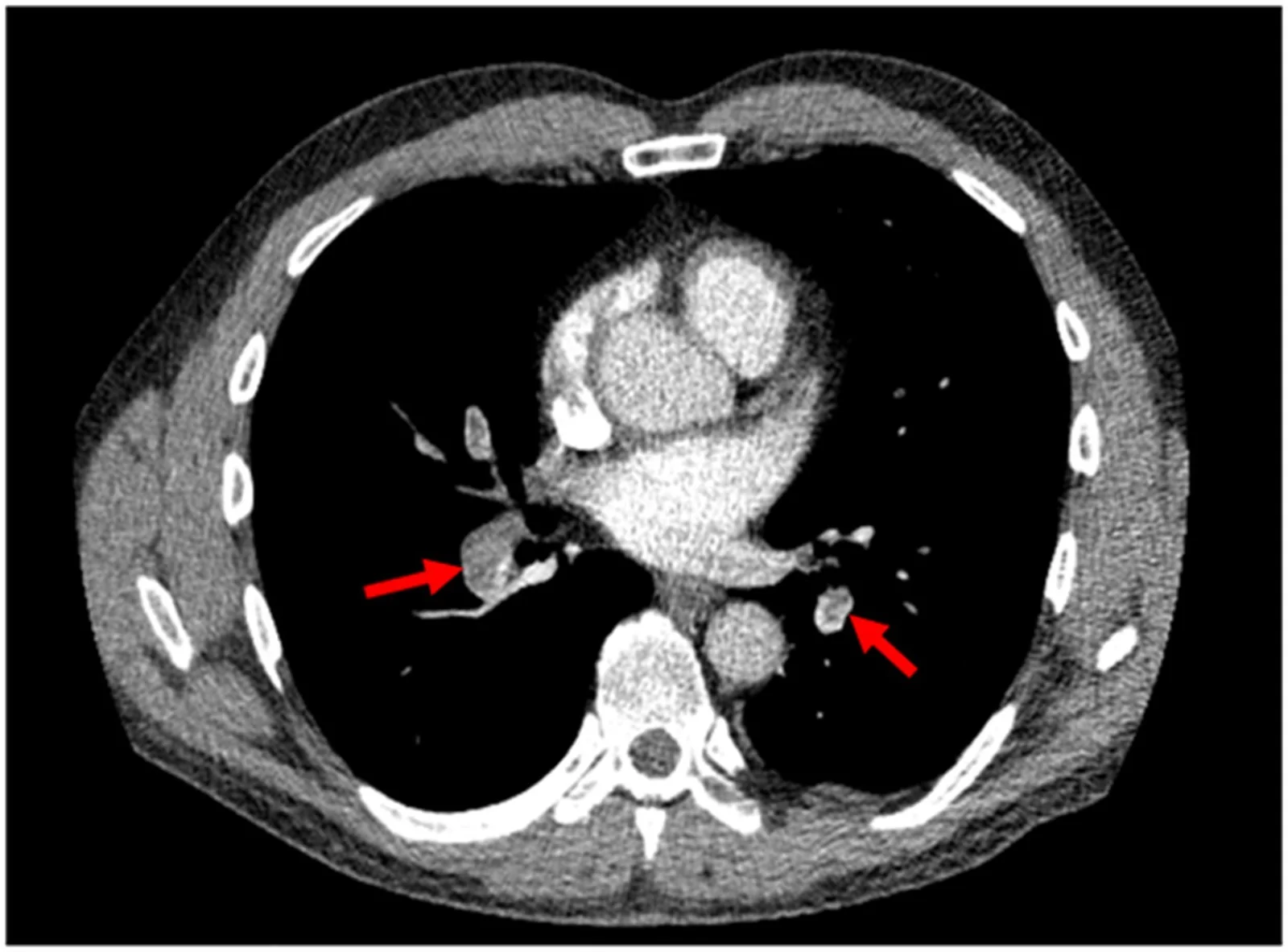
Thoracic CT scan demonstrating bilateral segmental and subsegmental pulmonary emboli.

A cardiology consultation was obtained, and urgent echocardiography was performed. The examination was unremarkable, demonstrating normal right ventricular size and function. Troponin levels were not elevated.

Doppler ultrasonography of the lower extremities demonstrated no evidence of arterial or deep vein thrombosis. Electrocardiography was within normal limits.

Ongoing TRT, with self-shortened injection intervals, was considered a likely contributing factor to marked polycythemia and bilateral PE. Additional investigations were nevertheless performed to exclude relevant coexisting thrombotic risk factors. Hematological evaluation revealed no evidence of an underlying thrombophilia (Table 1), while CT imaging of the thorax and abdomen showed no evidence of active malignancy. Further etiological evaluation was individualized based on the clinical context and the potential implications of additional findings for subsequent management.

Furthermore, comorbidities that may contribute to TRT-associated increases in hematocrit, including chronic obstructive pulmonary disease, obstructive sleep apnea, cardiovascular disease, and smoking, were excluded.

## Treatment

Based on risk stratification for management of PE, initial anticoagulation therapy with a direct oral anticoagulant was initiated. Due to a left-sided pulmonary infarction and the presence of leukocytosis and elevated CRP, empiric antibiotic therapy (amoxicillin–clavulanate) was started.

Testosterone replacement therapy, considered the likely cause of secondary polycythemia and a contributing risk factor for PE, was discontinued. In addition, therapeutic phlebotomy was scheduled.

## Outcome and follow-up

Four months later, the patient presented to our endocrinology outpatient clinic for follow-up. He reported a good general condition, with resolution of fatigue. Laboratory follow-up demonstrated normalization of Hb and testosterone levels following discontinuation of TRT.

Further multidisciplinary follow-up with cardiology, hematology, and urology was recommended.

## Discussion

Several observational studies have reported an increased risk of major adverse cardiovascular events associated with TRT, prompting the US Food and Drug Administration to issue a general warning regarding the potential risk of VTE associated with testosterone products [8]. However, current evidence regarding the association between TRT and VTE, particularly PE, remains controversial. These inconsistencies are likely attributable to the limited availability of evidence derived from randomized controlled trials [4].

The proposed mechanisms underlying the increased thrombotic risk associated with TRT include testosterone-induced erythropoiesis, leading to elevated hematocrit levels and increased blood viscosity. In addition, exogenous testosterone may promote a prothrombotic state through enhanced platelet aggregation, increased thromboxane A2 production, and augmented thrombin generation [9].

Current European guidelines recommend screening for secondary polycythemia during TRT in addition to assessing for active malignancy and evaluating the patient's cardiovascular risk profile. Furthermore, the absence of clinical improvement in hypogonadal symptoms should prompt reconsideration of the diagnosis and may warrant discontinuation of TRT and investigation of alternative etiologies [10].

In the present case, the historical diagnosis of primary hypogonadism had initially been attributed to unilateral orchiectomy. However, the patient's treatment history also included 4 cycles of adjuvant BEP chemotherapy following orchiectomy, providing an additional potential contributor to impaired gonadal function. Although unilateral orchiectomy alone is generally not expected to result in persistent testosterone deficiency because of compensatory Leydig cell function in the remaining testis, cisplatin-based chemotherapy has been associated with impaired Leydig cell function and an increased risk of testosterone deficiency in testicular cancer survivors [11-13]. A systematic review and meta-analysis demonstrated that standard platinum-based chemotherapy of up to 4 cycles was associated with a significantly increased risk of testosterone deficiency compared with orchiectomy alone [11]. Furthermore, longitudinal studies have demonstrated alterations in Leydig cell function following BEP chemotherapy, with a subset of patients developing persistent or progressive gonadal dysfunction and requiring testosterone replacement [12, 13]. Thus, the patient's previous gonadal dysfunction may plausibly have reflected the combined effects of unilateral orchiectomy and chemotherapy-related impairment of the remaining testicular function rather than unilateral orchiectomy alone.

Importantly, review of the original medical records confirmed testosterone concentrations below the reference range at the time hypogonadism was diagnosed and TRT was initiated, supporting the original biochemical diagnosis of testosterone deficiency. The subsequent normalization of serum testosterone after discontinuation of TRT therefore does not necessarily contradict the initial diagnosis but indicates preserved or recovered endogenous Leydig cell function at the time of follow-up.

This clinical course highlights the importance not only of establishing biochemical evidence of hypogonadism before initiating TRT but also of periodically reassessing endogenous gonadal function and the continued indication for TRT.

In the present case, the patient was hemodynamically and cardiopulmonary stable, with no classical clinical manifestations of PE. The predominant symptoms were fatigue, which overlap with typical manifestations of male hypogonadism. The patient independently modified the intervals of testosterone injection therapy, attributing his symptoms to insufficient testosterone levels.

Further, this case represents an uncommon and diagnostically challenging presentation, as patients with bilateral PE typically present with dyspnea, cardiopulmonary instability, and abnormal arterial blood gas analysis, findings that were absent in this patient. A missed diagnosis of PE could have had potentially fatal consequences. In this clinical setting, the decision to perform a thoracic CT and measure D-dimer levels was prompted by refractory fatigue and marked polycythemia. However, in routine clinical practice, these findings alone do not constitute standard indications to promptly exclude PE by imaging.

Another challenging aspect highlighted by this case is the lack of a standardized risk-stratification system for assessing the risk of PE in hypogonadal men receiving TRT, in which both laboratory and clinical parameters are systematically evaluated and integrated.

This case underscores the importance of clinical awareness among both patients and healthcare providers for the early recognition of symptoms suggestive of PE in hypogonadal men receiving TRT. In these patients, nonspecific symptoms such as fatigue and reduced exercise tolerance may be misattributed to hypogonadism rather than recognized as manifestations of an acute thromboembolic event.

Although the diagnosis may be challenging, the evaluation of suspected PE should include assessment for polycythemia and careful consideration of testosterone serum levels as a potential contributing risk factor. Given the limited evidence regarding individualized thromboembolic risk in this population, future prospective studies are warranted to develop and validate risk-stratification models that can guide clinical decision-making and optimize patient safety during TRT in hypogonadal men.

## Learning points

- Pulmonary embolism may present with nonspecific symptoms, including persistent fatigue, in hypogonadal men receiving TRT, potentially mimicking persistent androgen deficiency and delaying diagnosis.
- In hypogonadal men receiving TRT who develop polycythemia, VTE should be considered after exclusion of other potential causes.
- Following unilateral orchiectomy and gonadotoxic chemotherapy, endogenous testosterone production may recover over time, warranting periodic reassessment of the continued indication for TRT.
- In the absence of validated thromboembolic risk-stratification tools, regular clinical and laboratory monitoring remains essential for early identification of potential thrombotic complications.

## Contributors

All authors made individual contributions to authorship. H.E.H., S.D.H., J.T., and E.V. were involved in the diagnosis and management of the patient and in the follow-up. H.E.H. collected and interpreted the clinical data, performed the literature review, and contributed to the conceptualization and drafting of the manuscript. All authors reviewed and approved the final version of the manuscript.

## Data Availability

Data sharing is not applicable to this article as no datasets were generated or analyzed during the current study.

## Abbreviations

ALT: alanine aminotransferase
AST: aspartate aminotransferase
CRP: C-reactive protein
BEP: bleomycin, etoposide, and cisplatin
CT: computed tomography
Hb: hemoglobin
HDL: high-density lipoprotein
LDL: low-density lipoprotein
PE: pulmonary embolism
TRT: testosterone replacement therapy
TSH: thyroid-stimulating hormone
VTE: venous thromboembolism

## References

[1] Saad F, Aversa A, Isidori AM, Zafalon L, Zitzmann M, Gooren L. Onset of effects of testosterone treatment and time span until maximum effects are achieved. Eur J Endocrinol. 2011;165(5):675‐685. 10.1530/EJE-11-0221 PMC3188848 21753068

[2] Bhasin S, Brito JP, Cunningham GR, et al Testosterone therapy in men with hypogonadism: an endocrine society clinical practice guideline. J Clin Endocrinol Metab. 2018;103(5):1715‐1744. 10.1210/jc.2018-00229 29562364

[3] Lunenfeld B, Mskhalaya G, Zitzmann M, et al Recommendations on the diagnosis, treatment and monitoring of testosterone deficiency in men. Aging Male. 2021;24(1):119‐138. 10.1080/13685538.2021.1962840 34396893

[4] Cannarella R, Gusmano C, Leanza C, et al Testosterone replacement therapy and vascular thromboembolic events: a systematic review and meta-analysis. Asian J Androl. 2024;26(2):144‐154. 10.4103/aja202352 PMC10919420 37921515

[5] Lee K, Toraby S, Cotterman R, Oriowo B, Fish J. A tumultuous course of exogenous testosterone by a bodybuilder causing a catastrophic hypercoagulable state in the surgical intensive care unit. Case Rep Vasc Med. 2019;2019:3097865. 10.1155/2019/3097865 PMC6925774 31886016

[6] Liljeqvist S, Helldén A, Bergman U, Söderberg M. Pulmonary embolism associated with the use of anabolic steroids. Eur J Intern Med. 2008;19(3):214‐215. 10.1016/j.ejim.2007.03.016 18395167

[7] Nguyen SM, Ko Ko N, Sattar AS, Gucuk Ipek E, Ali S. Pulmonary embolism secondary to testosterone-enhancing herbal supplement use. Cureus. 2017;9(8):e1545. 10.7759/cureus.1545 PMC5630464 29018642

[8] Lokeshwar SD, Bitran J, Ramasamy R. Changes in testosterone prescribing patterns after FDA warning. Transl Androl Urol. 2019;8(S3):S287‐S288. 10.21037/tau.2019.04.03 PMC6642974 31392147

[9] Glueck CJ, Goldenberg N, Wang P. Testosterone therapy, thrombophilia, venous thromboembolism, and thrombotic events. J Clin Med. 2019;8(1):11. 10.3390/jcm8010011 PMC6352146 30577621

[10] Corona G, Goulis DG, Huhtaniemi I, et al European Academy of Andrology (EAA) guidelines on investigation, treatment and monitoring of functional hypogonadism in males: endorsing organization: European Society of Endocrinology. Andrology. 2020;8(5):970‐987. 10.1111/andr.12770 32026626

[11] Bandak M, Jørgensen N, Juul A, et al Testosterone deficiency in testicular cancer survivors—a systematic review and meta-analysis. Andrology. 2016;4(3):382‐388. 10.1111/andr.12177 27009402

[12] Bandak M, Jørgensen N, Juul A, et al Longitudinal changes in serum levels of testosterone and luteinizing hormone in testicular cancer patients after orchiectomy alone or bleomycin, etoposide, and cisplatin. Eur Urol Focus. 2018;4(4):591‐598. 10.1016/j.euf.2016.11.018 28753832

[13] Eberhard J, Ståhl O, Cwikiel M, et al Risk factors for post-treatment hypogonadism in testicular cancer patients. Eur J Endocrinol. 2008;158(4):561‐570. 10.1530/EJE-07-0684 18362304

